# Microbial acetone oxidation in coastal seawater

**DOI:** 10.3389/fmicb.2014.00243

**Published:** 2014-05-26

**Authors:** Joanna L. Dixon, Rachael Beale, Stephanie L. Sargeant, Glen A. Tarran, Philip D. Nightingale

**Affiliations:** Plymouth Marine Laboratory, Prospect PlacePlymouth, UK

**Keywords:** bacteria, kinetics, acetone oxidation, Western English Channel (L4), radioactive labeling, seasonality, acetone turnover

## Abstract

Acetone is an important oxygenated volatile organic compound (OVOC) in the troposphere where it influences the oxidizing capacity of the atmosphere. However, the air-sea flux is not well quantified, in part due to a lack of knowledge regarding which processes control oceanic concentrations, and, specifically whether microbial oxidation to CO_2_ represents a significant loss process. We demonstrate that ^14^C labeled acetone can be used to determine microbial oxidation to ^14^CO_2_. Linear microbial rates of acetone oxidation to CO_2_ were observed for between 0.75-3.5 h at a seasonally eutrophic coastal station located in the western English Channel (L4). A kinetic experiment in summer at station L4 gave a *V*_max_ of 4.1 pmol L^-1^ h^-1^, with a *K*_m_ constant of 54 pM. We then used this technique to obtain microbial acetone loss rates ranging between 1.2 and 42 pmol L^-1^ h^-1.^(monthly averages) over an annual cycle at L4, with maximum rates observed during winter months. The biological turnover time of acetone (*in situ* concentration divided by microbial oxidation rate) in surface waters varied from ~3 days in February 2011, when *in situ* concentrations were 3 ± 1 nM, to >240 days in June 2011, when concentrations were more than twofold higher at 7.5 ± 0.7 nM. These relatively low marine microbial acetone oxidation rates, when normalized to *in situ* concentrations, suggest that marine microbes preferentially utilize other OVOCs such as methanol and acetaldehyde.

## INTRODUCTION

Acetone is a ubiquitous oxygenated volatile organic compound (OVOC) in the troposphere [e.g., Singh et al., 1995, 2003; Lewis et al., 2005], and is thought to play an important role in the chemistry of the atmosphere by sequestering nitrogen oxides, and by providing HO_x_ radicals through photolysis (Singh et al., 1995; Wennberg et al., 1998), thus influencing the oxidizing capacity and ozone formation (Singh et al., 2001). The composition of OVOCs in the troposphere and lower stratosphere is dominated by acetone, acetaldehyde, and methanol, e.g., Read et al. (2012). Total global sources of acetone range between 37 and 95 million tons per year (Singh et al., 2000, 2001, 2004; Jacob et al., 2002). Primary terrestrial, e.g., pasture and forest emissions and secondary anthropogenic sources (including biogenic propane oxidation) account for approximately half of known acetone sources (Singh et al., 2000). The oceans are thought to play a major role in controlling atmospheric acetone levels (Fischer et al., 2012), although whether the oceans currently act as a net source or sink to the atmosphere is not clear (Williams et al., 2004; Lewis et al., 2005; Marandino et al., 2005; Taddei et al., 2009; Fischer et al., 2012). However, recent data suggest that the North and South oligotrophic gyres of the Atlantic Ocean are a source of acetone to the atmosphere, whilst near air–sea equilibrium conditions dominates over equatorial waters, and temperate open ocean regions (high northern and southern latitudes) show a flux from the atmosphere to the oceans (Beale et al., 2013).

Acetone is thought to be produced photochemically in seawater from chromophoric dissolved organic matter (Mopper and Stahovec, 1986; Kieber et al., 1990; Mopper et al., 1991; de Bruyn et al., 2011; Dixon et al., 2013a), with strong diurnal variability (Zhou and Mopper, 1997). Acetone production due to photochemical processes was recently estimated at 48–100% of gross production for remote Atlantic Ocean surface waters (Dixon et al., 2013a). Biological production of substantial amounts of acetone (up to 8.7 mM) by cultured marine *Vibrio* species during degradation of leucine has also been reported (Nemecek-Marshall et al., 1995). Acetone is also an intermediate in the metabolism of propane, and is converted, via acetol to either acetaldehyde (+formaldehyde), acetic acid (+formaldehyde) or ultimately to pyruvic acid by a number of bacteria such as *Rhodococcus* and *Mycobacterium*. As both of these species are widespread in terrestrial and marine environments (Hartmans and de Bont, 1986; Ashraf et al., 1994), biological production of acetone is considered likely in agreement with recent marine incubation experiments (Dixon et al., 2013a).

Acetone losses in seawater are less well understood. Previous bacterial culture experiments have shown microbial uptake of acetone (Rathbun et al., 1982; Sluis and Ensign, 1997) with insignificant losses due to direct photolysis in fresh and riverine waters (Rathbun et al., 1982). Loss of acetone in seawater samples from a coastal station in the Pacific Ocean (33.6N, 118W) have recently suggested a short half-life of 5.8 ± 2.4 h with significant diurnal and seasonal variability (higher loss rates observed during winter and earlier in the day, de Bruyn et al., 2013). However, this contrasts with estimates from surface open ocean Atlantic waters where a comparison of *in situ* acetone concentrations with microbial oxidation rates from incubation experiments suggest much longer biological lifetimes ranging between 3 and 82 days (Beale et al., 2013; Dixon et al., 2013a). Acetone oxidation rates have been shown to linearly positively correlate with bacterial production (Dixon et al., 2013a), and an inverse linear relationship has also been observed between acetone seawater concentrations and bacterial production (Beale et al., 2013). Thus, despite relatively low microbial acetone oxidation rates (compared to other OVOCs like methanol and acetaldehyde, Dixon et al., 2011a, b, 2013a; Dixon and Nightingale, 2012) these relationships suggest that as bacterial production increases, so does the rate of microbial acetone oxidation, leading to a reduction in the *in situ* concentration of acetone.

The aim of this study was to make a comprehensive assessment of the range and significance of microbial acetone oxidation rates over an annual cycle at a coastal observatory situated in the western English Channel.

## MATERIALS AND METHODS

We have used a radiochemical technique with pico-molar additions of ^14^C labeled acetone (^14^CH_3_CO^14^CH_3_) to seawater to determine the microbial transformation (oxidation) of acetone to carbon dioxide, in a similar approach to that of Dixon et al. (2011a) for ^14^C labeled methanol.

### SAMPLE COLLECTION

Surface water samples (≤10 m) were collected from a long term monitoring station, situated approximately 10 nautical miles south-west of Plymouth, called L4 (50.3N, 04.22W, water depth ~55 m, Smyth et al., 2010). Samples were pumped directly into acid-washed quartz Duran bottles and stored in the dark for the 2–3 h transit back to the laboratory. Labeled ^14^C acetone was purchased from American Radiolabeled Chemicals, Inc with a specific activity of 30 Ci mmol^-1^ (ARC0469, neat liquid in sealed ampoule). Primary stocks were made by diluting 1 mCi into 40 mls of 18 MΩ Milli Q water (0.025 mCi mL^-1^) and were stored in gas-tight amber vials in the dark at 4°C. Stability and storage trials suggested a loss in activity of <5% over 12 months. Addition volumes of ^14^C acetone to seawater samples were always <1% of the sample volume and typically ≤5% of the label was used during incubations ≤3.5 h.

### TIME COURSE EXPERIMENTS

**FIGURE 1:**
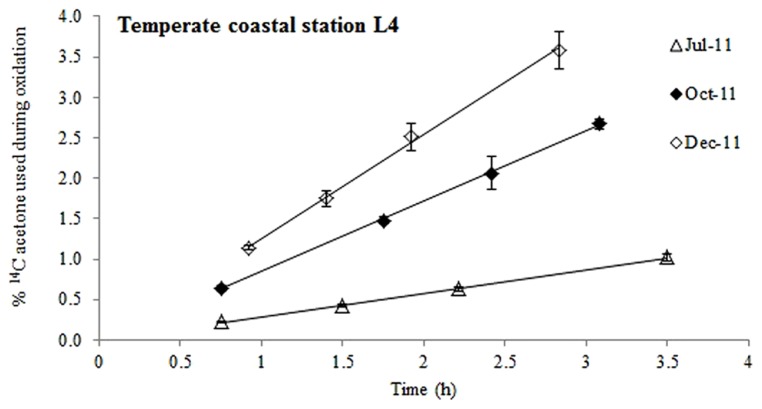
**Time course experiments showing the % of added ^14^C-labeled acetone that was used during oxidation at station L4 during different months of the year.** The error bars represent ±1 standard deviation based on three replicates.

Time course experiments were initially carried out to determine the period of linear incorporation of the ^14^C label. Labeled acetone (^14^C) was added to seawater samples to yield final concentrations of 40–90 pM (2700–6100 disintegrations per minute mL^-1^) depending on the experiment (**Figure 1**). Samples were incubated in acid washed polycarbonate bottles in the dark for between <1-6.5 h at *in situ* sea surface temperature. At selected times, triplicate sub-samples were taken to assess microbial oxidation to ^14^CO_2_. Oxidation of ^14^C labeled acetone to ^14^CO_2 _was determined by pipetting 1 ml samples into 2 ml micro centrifuge tubes and adding 0.5 ml of SrCl_2_.6H_2_O (1 M), to precipitate the ^14^CO_2_ as Sr^14^CO_3_, 20 μl of NaOH (1 M), to neutralize the HCl produced, and 100 μl of Na_2_CO_3 _(1 M), to ensure adequate pellet formation (Connell et al., 1997; Goodwin et al., 1998). After centrifugation the supernatant was aspirated, the pellet washed twice with ethanol (80%), resuspended in 1 ml of concentrated NaOH solution (~ 10 nM) that had been adjusted to a pH of 11.7, before addition of Optiphase HiSafe III to create a slurry. The samples were vortex mixed and stored in the dark for >24 h before being analyzed on a scintillation counter (Tricarb 3100 or 2910, Perkin Elmer). This period ensures that any chemiluminescence arising from interactions between NaOH and Optiphase scintillant subside (Kiene and Hoffmann Williams, 1998).

### KINETIC DETERMINATIONS

The kinetics of microbial acetone oxidation were investigated at L4 during February and June 2011 using 1.0 ml surface seawater samples. Surface samples received an addition of ^14^C-labeled acetone, and a series of tubes for microbial oxidation were treated to yield a range of ^14^C concentrations between 2 and 47 nM (~2.5% of added ^14^C acetone was oxidized) during February and between 6 and 1006 pM (1.4–5.5% of added ^14^C acetone was oxidized) during June 2011. Samples were incubated in screw topped, O-ring sealed micro tubes in the dark at *in situ* temperature. Three replicates from each acetone concentration were processed, as detailed above, after approximately 1 h incubation period.

### ACETONE OXIDATION RATES

Triplicate seawater samples (1 ml) were amended with ^14^C labeled acetone as detailed previously. Microbial acetone oxidation rates (pmol L^-1^ h^-1^) were calculated by multiplying the sample counts (nCi mL^-1^ h^-1^, where 1 Ci = 3.7 × 10^10 ^Bq) by the specific activity of ^14^C acetone (30 Ci mmol^-1^). All rates were corrected by subtracting killed sample counts (Trichloroacetic acid, TCA, 5% final concentration) to correct for non-biological processes. TCA is regularly used for killed controls, e.g., when measuring bacterial production indirectly via ^3^H-leucine incorporation (Smith and Azam, 1992), and does not lyse cells.

### SEAWATER ACETONE CONCENTRATIONS

Surface seawater was collected in Niskin bottles, and transferred into brown glass sample bottles with gas-tight stoppers using Tygon^TM^ tubing. Acetone concentrations were determined using a membrane inlet system coupled to a proton transfer reaction mass spectrometer (Beale et al., 2011).

### BACTERIAL PRODUCTION, CHLOROPHYLL A CONCENTRATION, AND COMMUNITY COMPOSITION

Rates of bacterial protein production (BP) and the numbers of heterotrophic bacteria, *Synechococcus* spp and picoeukaryotes were also determined to investigate any trends. BP was determined by measuring the incorporation of ^3^H-leucine (20 nM final concentration) into bacterial protein on 1.7 ml seawater samples following the method of Smith and Azam (1992). The numbers of bacterioplankton cells were determined by flow cytometry on SYBR Green I DNA-stained cells from 1.8 ml seawater samples fixed in paraformaldehyde (0.5–1%, final concentration), flash frozen in liquid nitrogen immediately after fixation, and stored frozen at -80°C (Marie et al., 1997). Numbers of *Synechococcus* spp and picoeukaryotes were analyzed on unstained samples by flow cytometry (Zubkov et al., 2000). Chlorophyll a samples were determined by fluorometric analysis of acetone-extracted pigments (Holm-Hansen et al., 1965).

## RESULTS

### LINEAR TIME COURSE EXPERIMENTS

When pico-molar concentrations of ^14^C labeled acetone were added to surface waters from station L4, radioactive carbon was expired to ^14^CO_2 _(**Figure 1**) suggesting that acetone was used as a microbial energy source. At this coastal station, acetone oxidation was linear for up to ~3.5 h, after which between 1 and 3.6% of the added label had been oxidized to ^14^CO_2_. Microbial acetone oxidation rates were highest in December 2011 (9.5 pCi mL^-1^ h^-1^, *R*^2^ = 0.997, *n* = 4) and lowest during July 2011 (2.5 pCi mL^-1^ h^-1^, *R*^2^ = 0.999, *n* = 4).

### UPTAKE KINETICS

**FIGURE 2:**
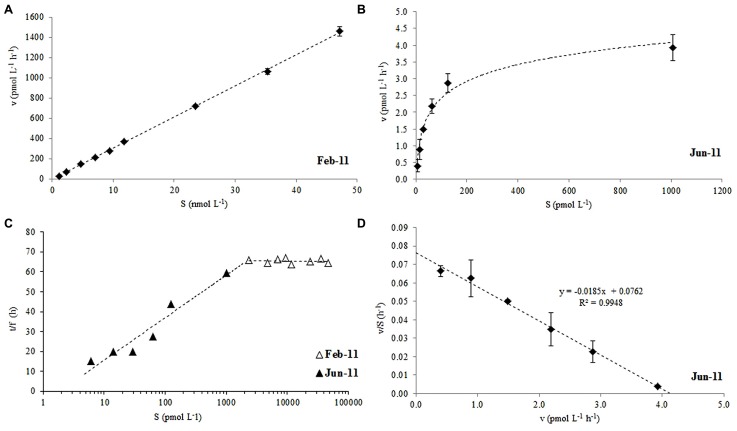
**Rate of acetone oxidation (v) against added substrate concentration (^14^C-labeled acetone, S) at **(A)** nano-molar additions in February, **(B)** pico-molar additions in June, **(C)** modified Lineweaver-Burke plot of combined Feb and June 2011 data and, **(D)** Eadie-Hofstee plot of June data used to derive *V*_**max**_and *K*_**m**_for surface waters of station L4.** For **(C)** the time of incubation (t) divided by the fraction of label oxidized to CO_2_ (f) is plotted against S. The error bars represent ±1 standard deviation based on three replicates.

The microbial oxidation of ^14^C labeled acetone displayed non-saturation type kinetics for nano-molar additions of acetone between 2 and 47 nmol L^-1^ during February 2011 (**Figure 2A**), which, when plotted as a modified Lineweaver-Burke plot (**Figure 2C**, △), showed a constant fraction of added label (*f* = 0.025 ± 0.001) had been oxidized to CO_2_, irrespective of the initial addition concentration. Pico-molar ^14^C-acetone additions (6-1006 pmol L^-1^) were made in the following June which resulted in saturation kinetics (**Figure 2B**), where the fraction of acetone oxidized reduced from 5.5 to 1.4% with increasing addition concentrations (**Figure 2C**; ▲). Saturation kinetics displayed during June 2011 allowed the first estimates of *V*_max_ and *K*_m_ to be determined from an Eadie-Hofstee plot (**Figure 2D**) of 4.1 pmol L^-1^ h^-1^ and 54 pmol L^-1^, respectively, for surface coastal waters of station L4.

### SURFACE SEASONAL TRENDS IN MICROBIAL ACETONE OXIDATION

**FIGURE 3:**
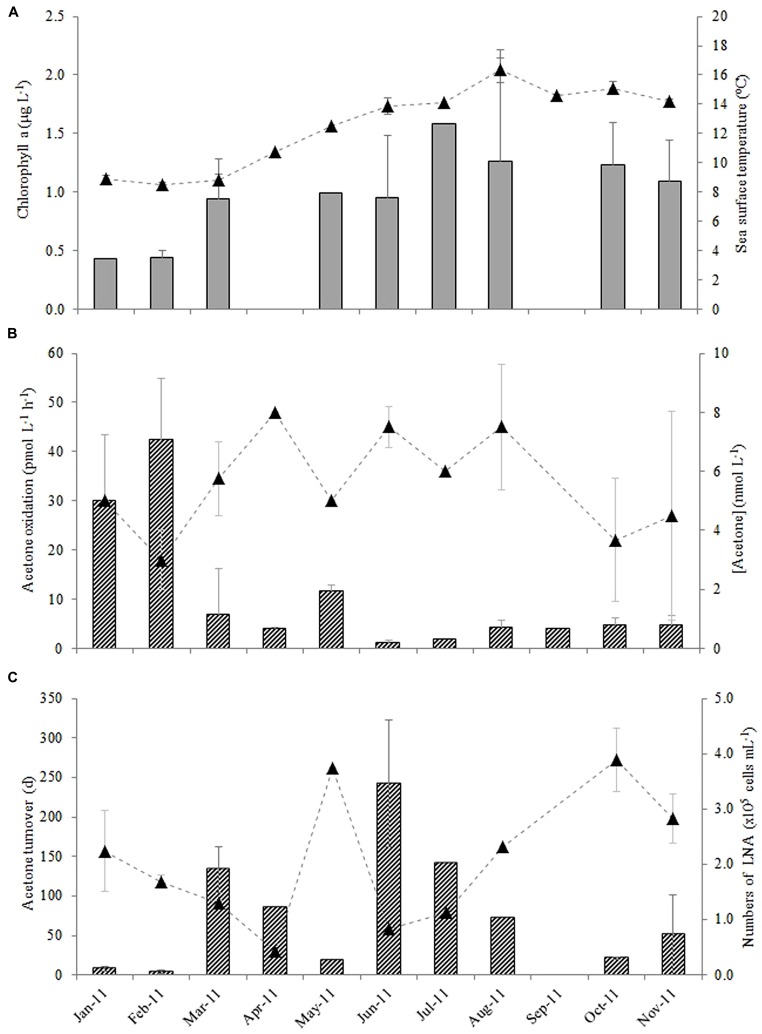
**Monthly variability in surface waters at station L4 for (A) chlorophyll a (bars) and sea surface temperature (▲), **(B)** acetone oxidation rates (bars) and *in situ* seawater acetone concentrations (▲) and **(C)** resulting microbial turnover times (bars) with corresponding changes in the numbers of low nucleic acid containing bacteria (▲, LNA where there is a significant linear correlation between the microbial turnover time of acetone and the numbers of low nucleic acid containing bacteria, *r* = -0.589, *n* = 16, *P* < 0.02).** The error bars represent ±1 standard deviation based on three replicates.

The average monthly rates of microbial oxidation of acetone in surface waters at station L4 varied between 1.2 and 42 pmol L^-1^ h^-1^ (**Figure 3B**) and showed significant changes with season. Oxidation rates were highest during winter (January and February 2011) at 36.2 ± 8.7 pmol L^-1^ h^-1^ and were 15-fold lower during the summer (June, July, and August 2011) at 2.4 ± 1.7 pmol L^-1^ h^-1^, with intermediate spring (March, April, May) and autumn (September, October, November) rates averaging 7.5 ± 4.0 and 4.5 ± 0.4 pmol L^-1^ h^-1^, respectively. When *in situ* seawater acetone concentrations are divided by microbial oxidation rates, biological turnover times are estimated, ranging between just over 3 days in February to ~243 days in June during 2011 (**Figure 3C**). This suggests a clear seasonal trend of longer microbial turnover times in spring and summer months compared to autumn and winter. Corresponding monthly averaged changes in low nucleic acid containing bacteria are also shown in **Figure 3C** ranging between 0.44 and 3.9 × 10^5^ cells mL^-1^, which show an opposite trend to microbial acetone turnover times (*r* = -0.589, *n* = 16, *P* < 0.02). Sea surface temperature at station L4 varied between 8.5 and 16.4°C, with typical low chlorophyll a values of ~0.4 μg L^-1^ during winter months rising fourfold to 1.6 μg L^-1^ in July 2011 (**Figure 3A**). Additionally, average monthly numbers of high nucleic acid containing bacteria (1.3–5.8 × 10^5 ^cells mL^-1^), *Synechococcus* sp. (0.7–36 × 10^3 ^cells mL^-1^), pico- (0.6–16 × 10^3 ^cells mL^-1^), and nano- (0.2–1.5 × 10^3 ^cells mL^-1^), phytoplankton cell, and bacterial leucine incorporation rates (8–96 pmol leucine L^-1 ^h^-1^), are summarized in **Table 1**.

**Table 1:** Summary of sampling at coastal station L4.

Month 2011	SST^a^ (°C)	Chl a^b^ (μg L^ - 1^)	LNA bacteria^c^ (×10^ 5^ cells mL^ - 1^)	HNA bacteria^d^ (×10^ 5^ cells mL^ - 1^)	Syns^e^ (×10^ 3^ cells mL^ - 1^)	Peuks^f^ (×10^ 3^ cells mL^ - 1^)	Nano^g^ (×10^ 3^ cells mL^ - 1^)	BP^h^ (pmol Leu L^ - 1^ h^ - 1^)
**Winter**
January	8.9 ± 0.3	0.43	2.2 ± 0.73	2.2 ± 0.70	9.3 ± 0.14	5.8 ± 0.77	0.41 ± 0.08	-
February	8.5 ± 0.2	0.44 ± 0.06	1.7 ± 0.1	1.7 ± 0.40	12.5 ± 2.4	8.9 ± 2.5	0.22 ± 0.04	-
**Spring**
March	8.9 ± 0.4	0.94 ± 0.34	1.3 ± 0.1	1.3 ± 0.02	5.0 ± 2.3	7.9 ± 0.31	1.0 ± 0.46	-
April	10.8	-	0.44	1.8	0.74	3.0	1.3	28.6
May	12.5	0.99	3.7	5.8	2.8	26.7	3.9	61.4
**Summer**
June	13.8 ± 0.6	0.95 ± 0.54	0.83 ± 0.05	4.4 ± 0.4	2.7 ± 2.3	9.7 ± 1.9	1.5 ± 0.44	49.9 ± 30.3
July	14.1	1.59	1.1	2.3	3.5	3.7	0.97	96.2
August	16.4 ± 0.8	1.26 ± 0.96	2.3	4.4	27.0	0.63	0.60	52.0 ± 23.5
**Autumn**
September	14.6 ± 0.1	-	-	-	-	-	-	-
October	15.1 ± 0.4	1.24 ± 0.36	3.9 ± 0.6	3.4 ± 0.72	35.9 ± 9.8	15.8 ± 1.9	1.1 ± 0.09	8.0 ± 0.02
November	14.2 ± 0.1	1.1 ± 0.3	2.8 ± 0.5	2.6 ± 0.09	13.1 ± 5.3	7.2 ± 0.86	0.72 ± 0.06	10.2 ± 6.2

*All samples were collected from the surface (≤10 m). ^a^Sea surface temperature, ^b^Surface concentration of chlorophyll a, ^c^Number of low nucleic acid containing bacteria, ^d^Number of high nucleic acid containing bacteria, ^e^*Synechococcus* sp., ^f^Picophytolankton (<2 μm), ^g^Nanophytolankton (~2-12 μm), ^h^Bacterial production, When there is >1 sampling date contributing to the monthly average, ± 1 SD is quoted. All parameters except BP were obtained from the L4 database, which is provided by the Plymouth Marine Laboratory, Western Channel Observatory.*

### DEPTH VARIABILITY IN MICROBIAL ACETONE OXIDATION

**FIGURE 4:**
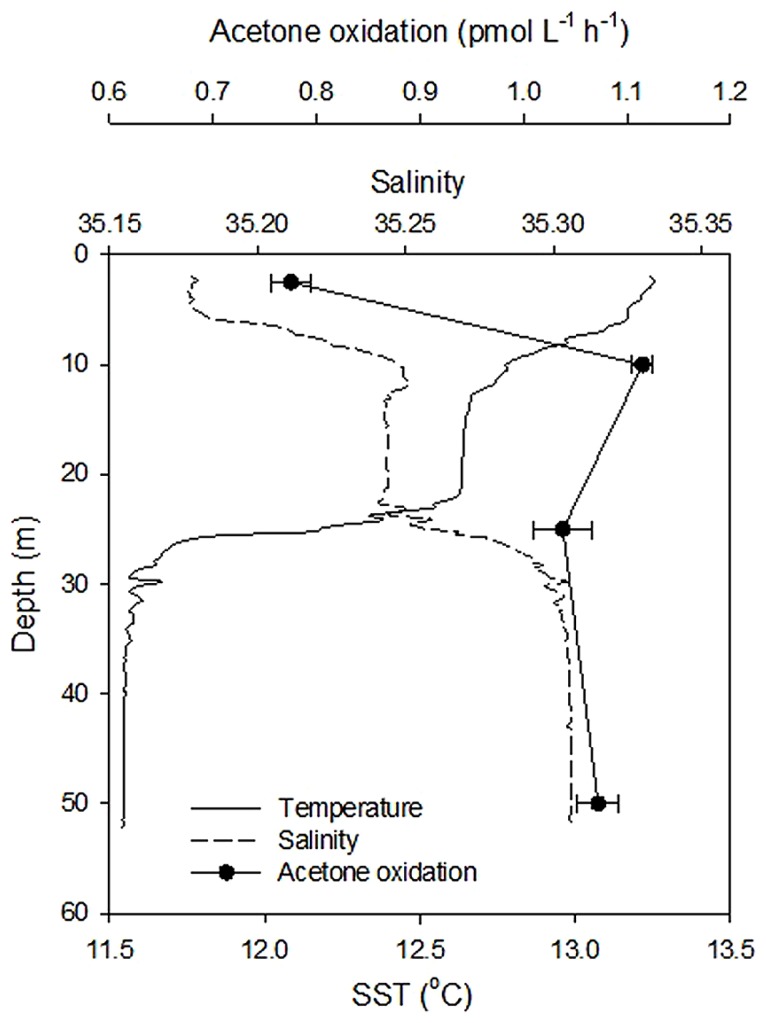
**Variability in acetone oxidation rates at coastal station L4 with depth during June 2011.** The error bars represent ± 1 standard deviation based on three replicates.

The variability of microbial acetone oxidation rates with depth at the relatively shallow (~55 m) coastal station L4 was investigated during June 2011, when surface rates were at their lowest, but the water column was seasonally stratified (see **Figure 4**). Microbial acetone oxidation rates were lowest (0.78 ± 0.02 pmol L^-1^ h^-1^) in the shallow surface layer (<10 m), which showed enhanced surface warming and relatively lower salinity. Rates were on average, more than 30% higher at greater depths (average of 1.07 ± 0.04 pmol L^-1^ h^-1^).

## DISCUSSION

This study shows that ^14^C labeled acetone can be used successfully to determine microbial oxidation rates (to ^14^CO_2_) in seawater samples. We report the first estimates of *V*_max_ (4.1 pmol L^-1^ h^-1^) and *K*_m_ (54 pmol L^-1^) for surface coastal waters during summer, when *in situ* surface oxidation rates were at their lowest (1.2 ± 0.39 pmol L^-1^ h^-1^, **Figure 3B**), despite relatively high average *in situ* acetone concentrations of 7.5 ± 0.7 nmol L^-1^. When nano-molar (2–47 nM) ^14^C acetone additions were made during winter months, first order kinetics were observed, but **Figure 2C** shows that a constant fraction of added label was oxidized to CO_2_, suggesting that any microbial enzyme systems involved in the conversion of acetone to CO_2_ were saturated. Pico-molar additions made during the summer, when acetone concentrations had more than doubled, showed first order reaction kinetics for approximately <100 pM acetone additions (**Figure 2B**). Both sets of data combined in a modified Lineweaver-Burke plot (**Figure 2C**, which assumes that if pico-molar additions had been made during winter, similar first order kinetics to summer would be observed) suggest *in situ* enzyme system saturation of 1–2 nM of mixed natural communities. Although the microbial composition of surface waters at L4 are highly likely to be different between the two seasons (e.g., Gilbert et al., 2009, 2012), it is unknown which microbes actively respire acetone to CO_2_. However, it is noteworthy that seasonal changes in bacterial structure have been linked to change in day length (Gilbert et al., 2012) and other environmental variables (e.g., temperature, Gilbert et al., 2009) rather than trophic interactions.

The microbial acetone oxidation kinetics observed during February for nano-molar additions does not show rate limitation with increasing substrate concentration, and thus does not comply with Michaelis–Menten kinetics (Wright and Hobbie, 1966), which could indicate no active microbial enzyme transport systems for acetone oxidation. These authors also showed that the slope of such a linear relationship between uptake rates and added substrate concentration (as in **Figure 2A**) was identical to the kinetics of simple diffusion. In addition, when samples were killed with TCA (5% final concentration), acetone oxidation did not increase over time, suggesting that, despite a possible lack of active transport systems, the uptake was nevertheless due to microbial metabolic activity. Wright and Hobbie (1966) suggested that at very low concentrations of added substrate, most glucose was incorporated using active bacterial transport systems, while at higher concentrations diffusion across algal cells dominated. Our results suggest that when pico-molar additions are made (June 2011) active transport systems dominated with a resultant mixed community *V*_max_ of 4.1 pmol L^-1^ h^-1^ and a *K*_m_ of 54 pmol L^-1^. However when nano-molar additions are made (February 2011) non saturation kinetics were observed, with possible diffusion across cell walls dominating (*cf.* methanol Dixon et al., 2011a).

Acetone oxidation by natural marine microbial communities could also be due to mixotrophic and heterotrophic phytoplankton in addition to heterotrophic bacteria. For rates of microbial acetone oxidation during February, which increased linearly with substrate concentration (*y* = 0.031x - 0.003, *n* = 9, *R*^2 ^= 0.999 for 1.7 h incubation period, **Figure 2A**) a diffusion constant (*K*_d_) can be calculated from the slope of the linear relationship (Wright and Hobbie, 1965). This constant assumes that organisms oxidize the acetone as rapidly as it diffuses in (Wright and Hobbie, 1965). A *K*_d_ of 0.003 h^-1^ is equivalent to a turnover time of ~1.4 days (Wright and Hobbie, 1965) which is comparable to the average estimate of 3.2 days for February 2011 determined in **Figure 3C**. This also compares well with the turnover of other organic compounds like DMS (e.g., 0.3–2.1 days, Simó et al., 2000) and methanol (e.g., 7 days in productive shelf waters, Dixon et al., 2011a). Despite the faster (i.e., hours) estimated acetone turnover times of de Bruyn et al. (2013), they also reported higher loss rates during the winter compared to other times of the year. However, the acetone turnover times reported by de Bruyn et al. (2013) originate from riverine and very near-shore costal environments (average salinity of 25.8 ± 2.1), that experience much less seasonal variability (average surface temperature of 17.5 ± 1.2°C) and higher average *in situ* acetone concentrations (59 ± 56 nM) compared to L4 waters (average salinity of 35.2 ± 0.1, average surface temperature of 12.5 ± 2.8°C, average surface acetone concentrations of 5.6 ± 2.3 nM). Furthermore, de Bruyn et al. (2013) report higher acetone loss rates after rain events, which could suggest faster microbial removal associated with less saline waters, although this is not reflected in **Figure 4**.

Acetone production in seawater is largely thought to be a photochemical process (Kieber et al., 1990; Zhou and Mopper, 1997; de Bruyn et al., 2011; Dixon et al., 2013a), possibly related to UV breakdown of chromophoric dissolved organic matter (CDOM) originating from eukaryotic cells (Dixon et al., 2013a). Given the relatively high microbial acetone oxidation rates found during January/February 2011 (in this study and in de Bruyn et al., 2013), with turnover times estimated at 1.4–3.2 days, it is not presently understood what process maintains acetone levels during winter months, when average acetone concentrations are 3.4 ± 1.1 nM. Typically, during winter at L4, UV levels and phytoplankton biomass are relatively low (e.g., Smyth et al., 2010). However, the water column is fully mixed and more influenced by riverine waters, i.e., maximum river flows and re-suspension events of bottom sediments (Groom et al., 2009). Thus during these periods it is probable that the dissolved organic matter is dominated by terrestrial sources and re-suspended sediments rather than phytoplankton.

Relationships between microbial oxidation and turnover of acetone with other biogeochemical variables (see **Table 1**) have been explored, and reveal statistically significant negative linear relationships between acetone oxidation rates and both sea surface temperature and concentration of chlorophyll *a* (*r* = -0.604 and -0.543, respectively for *n* = 21, *P* ≤ 0.02). This is largely because the highest acetone oxidation rates, were found during winter when sea surface temperatures and phytoplankton biomass were at their minima.

A statistically significant inverse relationship was also found between biological acetone turnover times and the numbers of low nucleic acid bacteria (LNA, *r* = -0.589, *n* = 16, *P* < 0.02). As previously noted, we do not know which marine microbes are capable of utilizing acetone, or the enzyme system(s) involved in the conversion of acetone to CO_2_, but this relationship indicates that low nucleic acid containing bacteria could be responsible for marine acetone consumption in surface coastal waters. SAR11 *Alphaproteobacteria*, are often significant components of the LNA (Mary et al., 2006) and are the most abundant heterotrophs in the oceans. SAR11 cells are believed to play a major role in mineralizing dissolved organic carbon (Sun et al., 2011) being efficient competitors for resources (Morris et al., 2002). Whilst in culture, Sun et al. (2011) found that *Candidatus Pelagibacter ubique* (a subgroup of SAR11) have the genome encoded pathways for the oxidation of a variety of one-carbon compounds, including the OVOC compound methanol. We found that the SAR11 clade were the second most numerically dominant bacterial order of surface bacterial populations found at station L4 during the annual sampling period 2011–2012, and contributed between 16 and 46% during winter months (Sargeant, 2014). *Alphaproteobacteria* were also the most abundant bacterial Class found at station L4 over a 6 year study (Gilbert et al., 2012). This study further reported that members of the *Rickettsiales* (SAR11) and *Rhodobacteriales* were the most frequently recorded operational taxonomic units, with the abundance of *Rickettsiales* reaching a maxima in winter (Gilbert et al., 2012), coincident with relatively fast acetone turnover times of ~3 days, found in this study.

The acetone biological turnover times determined here should be considered as conservative, because it is possible that some heterotrophic bacteria also assimilate acetone carbon into particulate carbon biomass *cf.* methanol, Dixon et al. (2013b). Furthermore, microbial acetone uptake that gets transformed and excreted as more refractory DOC compounds (as in the microbial carbon pump, e.g., Ogawa et al., 2001; Jiao and Azam, 2011), possibly via some overflow metabolism strategies as previously suggested for methanol (Dixon et al., 2013a) will also not be revealed via the experimental approach of this study.

Coastal surface water microbial acetone oxidation rates have been normalized to *in situ* concentration as a function of season, and are compared to other biologically utilized OVOC compounds (acetaldehyde and methanol, e.g., Dixon et al., 2013a) in **Table 2**. Acetone is a less preferred organic compound for marine microbes compared to methanol and acetaldehyde, although acetone oxidation rates shows a much more pronounced seasonality. In addition, the one depth profile undertaken during summer suggests near-surface reduction in microbial acetone oxidation rates associated with a less saline, warmer tongue of water in the top 10 m.

**Table 2:** Surface microbial oxidation rates normalized to in situ concentration (h^-1^) and resulting turnover times, as a function of season for coastal station, L4.

Season^a^	Acetone		Acetaldehyde		Methanol	
	h^-1^	Days	h^-1^	Hours	h^-1^	Day
Winter	0.012 ± 0.007 (5)	3.5	0.86 ± 0.55 (4)	1.2	n/a	n/a
Spring	0.001 ± 0.001 (6)	42	0.87 ± 0.38 (6)	1.1	0.03 ± 0.03 (4)	1.3
Summer	0.0004 ± 0.0002 (5)	104	0.95 ± 0.50 (4)	1.1	0.06 ± 0.06 (4)	0.73
Autumn	0.002 ± 0.001 (5)	21	2.4 ± 2.5 (5)	0.4	0.16 ± 0.04 (5)	0.25

*Where the numbers in brackets denote number sampling dates. ^a^Winter is defined a December, January, February; Spring as March, April, May; Summer as June, July and August; Autumn as September, October, November during 2011. n/a data not available.*

The kinetic characteristics of microbial acetone oxidation can be compared to those of other substrates commonly used by bacteria, so that the ecological significance of acetone to marine microbial metabolism can be evaluated. Both *V*_max _and *K*_m _are more than 2 orders of magnitude smaller for acetone oxidation compared to methanol oxidation (Dixon et al., 2011a), which if compared further with proteins and carbohydrates gives the following order; proteins >>carbohydrates ≈ methanol>>acetone (refer to Dixon et al., 2011a for protein, carbohydrate, and methanol *V*_max_ and *K*_m_ data).

This research offers the first comprehensive seasonally resolved study combining microbial acetone oxidation rates with *in situ* concentrations in order to derive biological turnover times that ranged between ~3 days in winter to >240 days in summer. We have experimentally derived the first *V*_max_ and *K*_m_ estimates of microbial acetone oxidation. We have also highlighted that there must be an unrecognized production mechanism for acetone during winter in coastal regions, possibly relating in some way, to enhanced dissolved organic matter from terrestrial sources. Further research should investigate possible winter acetone production mechanisms, identify which microbial species are utilizing acetone in marine environments, and characterize what enzyme systems are involved in the oxidation process.

## Conflict of Interest Statement

The authors declare that the research was conducted in the absence of any commercial or financial relationships that could be construed as a potential conflict of interest.
